# Broad-range PCR, cloning and sequencing of the full 16S rRNA gene for detection of bacterial DNA in synovial fluid samples of Tunisian patients with reactive and undifferentiated arthritis

**DOI:** 10.1186/ar2748

**Published:** 2009-07-01

**Authors:** Mariam Siala, Radhouane Gdoura, Hela Fourati, Markus Rihl, Benoit Jaulhac, Mohamed Younes, Jean Sibilia, Sofien Baklouti, Naceur Bargaoui, Slaheddine Sellami, Abdelghani Sghir, Adnane Hammami

**Affiliations:** 1 Laboratoire de Recherche 'Micro-organismes et Pathologie Humaine', EPS Habib Bourguiba, Rue El Ferdaous, 3029 Sfax, Tunisie; 2 Service de Rhumatologie Hôpital Hedi Chaker, Avenue Majida Boulila, 3029 Sfax, Tunisie; 3 Hannover Medical School (MHH), Clinic for Immunology and Rheumatology, 30625 Hannover; Germany; 4 Laboratoire de Physiopathologie des Interactions Hôte-bactérie, UPRES-EA 3432, Faculté de Médecine, Université Louis-Pasteur, rue Koeberlé, 67000 Strasbourg, France; 5 Service de Rhumatologie, EPS Fattouma Bourguiba, Rue 1er Juin, 5019 Monastir, Tunisie; 6 Service de Rhumatologie, EPS La Rabta, rue 7051 Centre Urbain Nord, 1082 Tunis, Tunisie; 7 CNRS-UMR 8030, CEA-Genoscope, rue Gaston Crémieux, 91000 Évry, France; 8 University of Evry Val d'Essonne, Boulevard François Mitterrand, 91025 Évry Cedex, 91000 Évry, France

## Abstract

**Introduction:**

Broad-range rDNA PCR provides an alternative, cultivation-independent approach for identifying bacterial DNA in reactive and other form of arthritis. The aim of this study was to use broad-range rDNA PCR targeting the 16S rRNA gene in patients with reactive and other forms of arthritis and to screen for the presence of DNA from any given bacterial species in synovial fluid (SF) samples.

**Methods:**

We examined the SF samples from a total of 27 patients consisting of patients with reactive arthritis (ReA) (n = 5), undifferentiated arthritis (UA) (n = 9), rheumatoid arthritis (n = 7), and osteoarthritis (n = 6) of which the latter two were used as controls. Using broad-range bacterial PCR amplifying a 1400 bp fragment from the 16S rRNA gene, we identified and sequenced at least 24 clones from each SF sample. To identify the corresponding bacteria, DNA sequences were compared to the EMBL (European Molecular Biology Laboratory) database.

**Results:**

Bacterial DNA was identified in 20 of the 27 SF samples (74, 10%). Analysis of a large number of sequences revealed the presence of DNA from more than one single bacterial species in the SF of all patients studied. The nearly complete sequences of the 1400 bp were obtained for most of the detected species. DNA of bacterial species including *Shigella *species, *Escherichia *species, and other coli-form bacteria as well as opportunistic pathogens such as *Stenotrophomonas maltophilia *and *Achromobacter xylosoxidans *were shared in all arthritis patients. Among pathogens described to trigger ReA, DNA from *Shigella sonnei *was found in ReA and UA patients. We also detected DNA from rarely occurring human pathogens such as *Aranicola *species and *Pantoea ananatis*. We also found DNA from bacteria so far not described in human infections such as *Bacillus niacini*, *Paenibacillus humicus*, *Diaphorobacter *species and uncultured bacterium genera incertae sedis OP10.

**Conclusions:**

Broad-range PCR followed by cloning and sequencing the entire 16S rDNA, allowed the identification of the bacterial DNA environment in the SF samples of arthritic patients. We found a wide spectrum of bacteria including those known to be involved in ReA and others not previously associated with arthritis.

## Introduction

The actual pathogenic event initiating arthritis is largely unknown. For several forms of arthritis, an infectious etiology has been postulated [1-3]. In particular, reactive arthritis (ReA) is known to be triggered by a variety of bacteria. For *Salmonella*, *Yersinia*, and *Chlamydia*, a persistent infection has been hypothesized due to the intraarticular presence of bacterial antigens, DNA, and/or RNA [4-6]. There is also evidence that undifferentiated arthritis is a form of ReA ('forme fruste') possibly due to a preceding but asymptomatic infection [7,8]. PCR using universal 16S rRNA primers is a highly sensitive tool allowing detection of unknown, that is unsuspected, pathogens relating to all eubacterial species [9-11]. This tool has been used before in patients with ReA, undifferentiated arthritis (UA), and other arthropathies. However, in most studies, the PCR products were of sufficient length to determine the genus of the bacteria in the synovial samples, but were not long enough to identify the species level [12-14].

We previously used the broad-range PCR, cloning and sequencing the entire 16S rDNA and demonstrated the presence of a large number of bacterial DNA in the synovial tissue (ST) of patients with ReA, UA, and other arthropathies [15]. These bacterial DNA were mainly derived from commensals that are normally present in the skin and gut. We also detected DNA of specific bacterial groups that have not been detected in arthritis samples or in human infections so far, suggesting that these new bacteria possibly could have a pathogenic relevance, particularly with regard to the ST. The detection of such a variety of bacterial groups after cloning and near full-length 16S rDNA sequencing obtained in ST samples has raised the question if the identical bacterial DNA communities could reside in both the ST and the synovial fluid (SF) of matched arthritis patients [15]. In addition, the composition of bacterial DNA from ST samples has not been compared comprehensively with that of matched SF samples in arthritis patients. Besides, a detailed analysis of SF bacterial DNA and their comparison with those from corresponding ST samples might help to determine whether intraarticular bacterial DNA might change over time between the two synovial compartments.

As opposed to SF [12,16], the bacterial DNA communities in the ST are well documented [13,14,17-19] and to our knowledge, there is no study that has amplified the entire 16S rDNA from SF samples.

Accordingly, we now continue our study using PCR as well as cloning and sequencing of the entire 16S rDNA to identify any bacterial DNA potentially present in the SF samples of patients with ReA, UA, and other forms of arthritis who were also analyzed previously [15].

## Materials and methods

### Patients and synovial fluid samples

Twenty-seven patients with active arthritis and knee effusion gave informed consent and were included in the study, which was approved by the local ethical committees. ST samples of these patients were used in a previous study [15]. The clinical characteristics as well as technical aspects regarding prevention of contamination during sampling have recently been published in detail [15].

Among the 27 patients included in the study, five were diagnosed with ReA, and nine with undifferentiated arthritis (UA); we also included seven patients with rheumatoid arthritis (RA) and six with osteoarthritis (OA) who served as controls. In the current study, the 27 patients were identical to the 27 patients analyzed in our previously published study (from patient 2 to patient 28) [15]. The clinical features and demographic characteristics of the patients are summarized in Table 1. SF samples were aspirated by standard needle puncture, snap frozen in liquid nitrogen, and stored at -80°C until analysis.

**Table 1:** Demographic and clinical features of the study patients

Diagnosis (patients; n = 27)	Median disease duration, months (range)	Actual age or median age, years (range)	Sex ratio (M/F)	Clinical details
ReA (n = 5)	2.4 (1 to 6)	32.4 (20 to 50)	4:1	
1		22	M	Sexually acquired ReA; *Ct *IgG positive^a^, *Ct *IgA positive serology^b^
2		40	F	Sexually acquired ReA;
3		20	M	Sexually acquired ReA; *Ct *IgG positive serology^a^; *Ct *IgA positive serology^b^; B27+^d^
4		50	M	Sexually acquired ReA; *Ct *IgA positive serology^b^; *Ct *positive PCR^c^; B27+^d^
5		30	M	Sexually acquired ReA
UA (n = 9)	25 (2 to 60)	40 (22 to 59)	5:4	-
RA (n = 7)	66 (12 to 228)	44 (39 to 53)	2:5	-
OA (n = 6)	14 (12 to 24)	58 (44 to 70)	5:1	-

^a ^Serology was determined for *Ct*-IgG antibodies by Micro Immunofluorescence assay as described by Wang and Grayston [29]. ^b^Serology was determined for *Ct*-IgA antibodies by ELISA (Labsystems, Hilsinki, Finland). ^c^*Chlamydia *PCR in genital swabs was determined by Cobas Amplicor PCR assay (Roche Diagnostics Molecular Systems, Inc, CA, USA). ^d^HLA-B27 positivity was determined using a microcytotoxicity assay.

*Ct *= *Chlamydia trachomatis*; RA = rheumatoid arthritis; ReA = reactive arthritis; OA = osteoarthritis; UA = undifferentiated arthritis.

### Automated DNA extraction from synovial fluid and broad-range PCR amplification of 16S rRNA genes, cloning and sequencing of bacterial DNA

For 10 minutes, 500 μl of SF were centrifuged at 10,000 g. The whole SF cell pellet was subjected to DNA extraction using the MagNA Pure system (Roche Molecular Biochemicals, Meylan, France). Prior to the MagNA Pure extraction, 500 μl of lysis buffer (200 mM sodium chloride, 20 mM Tris hydrochloric acid, pH 8, 50 mM ethylenediaminetetraacetic acid and 1% SDS) and 25 μl of proteinase K (10 mg/ml) (Sigma, St Louis, MO, USA) were added to the SF cell pellet.

Bacterial 16S rDNA fragments were amplified from SF extracted DNA by broad-range PCR amplification using universal bacterial 16S rRNA gene-specific oligonucleotide primers Bac08 and Uni1390, as previously described [15]. Products of the expected size (approximately 1400 bp) were inserted into a vector using a cloning kit (pGEM-T vector; Promega, Madison, WI, USA). Sequencing and 16S rDNA analysis were performed as previously described [15].

### Data analysis

Statistical analysis was performed using SPSS 11.0 software (SPSS; Chicago, IL, USA). A *P *< 0.05 was considered statistically significant.

## Results

### PCR positivity by the broad-range PCR amplification system

PCR was positive in 20 of the 27 (74.10%) patients indicating the presence of bacterial 16S rDNA within the synovial samples. Of note, PCR was positive in five out of five (100%) patients with ReA and also in nine out of nine (100%) patients with UA. In the control group, bacterial 16S rDNA was amplified in three out of seven (43%) RA patients and in three of six (50%) OA patients. Accordingly, the presence of bacterial DNA within the SF samples was significantly higher in ReA and UA patients as compared with RA and OA patients (14 out of 14 (100%) vs 6 of 13 (46.2%); *P *= 0.006).

At least 24 individual clones were selected from each PCR product and sequenced. A summary of the total number of sequences analyzed in each patient is depicted in Table 2. Only full-length (1300 to 1400 bp) sequences of high quality were analyzed in detail. Most bacterial sequences had at least 97% sequence similarity with any known cultivated or uncultured bacteria. The percentage of similarity to the best fit sequence in the database, the accession number, and the sequence length of each probe are listed in Table 3.

**Table 2:** Details of bacterial species-derived DNA sequences identified in each patient*

Patient	Total number of bacterial DNA sequences	DNA sequences identified in each patient
ReA		
1	45	*10 × Stenotrophomonas maltophilia*, 7 × *Shigella *species, 6 × *Propionibacterium acnes*, 3 × *Ralstonia *species, 3 × *Shigella sonnei*, 3 × uncultured bacterium, 2 × *Bradyrhizobium elkanii*, 1 × uncultured γ proteobacterium, 1 × uncultured γ proteobacterium, 2 × uncultured *Sphingobacterium *species, 1 × *Aranicola *species, 1 × *Agrobacterium *species, 1 × *Burkholderia *species, 1 × *Escherichia coli*, 1 × *Paenibacillus humicus*, 1 × *Pantoea ananatis*, 1 × uncultured β proteobacterium.
2	47	13 × *Stenotrophomonas maltophilia*, 4× *Shigella *species, 3 × *Aranicola *species, 3 × *Ralstonia *species, 2 × β proteobacterium, 2 × γ proteobacterium, 2 × uncultured β proteobacterium, 1 × *Agrobacterium *species,, 1 × Bacteroidetes bacterium, 1 × *Escherichia coli*, 1 × *Escherichia *species, 1 × *Flavobacterium mizutaii*, 1 × *Pantoea ananatis*, 1 × *Paenibacillus humicus*, 1 × *Paenibacillus *species, 1 × *Propionibacterium acnes*, 3 × *Shigella sonnei *1 × *Streptococcus mitis*, 1 × swine manure bacterium, 1 × uncultured α proteobacterium, 1 × uncultured Bacteroidetes bacterium, 1 × uncultured *Flavobacterium *species, 1 × uncultured soil bacterium.
3	42	10 × *Shigella *species, 9 × *Stenotrophomonas maltophilia*, 3 × *Aranicola *species, 3 × uncultured bacterium,, 2 × *Escherichia *species, 2 × *Paenibacillus humicus*, 2 × *Streptococcus mitis*, 1 × *Achromobacter xylosoxidans*, 1 × *Bradyrhizobium elkanii*, 1 × Bacteroidetes bacterium, 1 × *Flavobacterium mizutaii*, 1 × γ proteobacterium, 1 × *Propionibacterium acnes*, 1 × *Shigella sonnei*, 1 × *Streptococcus mitis*, 1 × uncultured *Flavobacterium *species, 1 × uncultured *Methylococcaceae bacterium*, 1 × uncultured Bacteroidetes bacterium
4	49	*11 × Stenotrophomonas maltophilia*, 6 × γ proteobacterium, 5 × *Shigella *species, 5 × *Shigella sonnei*, 3 × *Aranicola *species, 3 × *Comamonas testosteroni*, 3 × uncultured bacterium, 2 × *Escherichia *species, 2× *Propionibacterium acnes*, 2 × uncultured *Flavobacterium *species, 2 × uncultured β proteobacterium, 1 × *Alcaligenes faecalis*, 1 × *Flavobacterium mizutaii*, 1 × *Paenibacillus humicus *1 × *Pelomonas saccharophila*, 1 × *Pseudomonas *species.
5	31	5 × *Escherichia coli*, 4 × *Stenotrophomonas maltophilia*, 3 × *Escherichia coli*, 2 × *Pseudomonas *species, 2 × *Shigella *species, 2 × *Sphingomonas faeni*, 1 × *Alcaligenes faecalis*, 1 × β proteobacterium, 1 × *Comamonas testosteroni*, 1 × *Diaphorobacter *species, 1 *× Delftia acidovorans*, 1 *×Flavobacterium mizutaii*, 1 × γ proteobacterium, 1 × uncultured bacterium, 1 × uncultured β proteobacterium, 1 × *Pantoea ananatis*, 1 × *Propionibacterium acnes*, 1 × *Ralstonia *species, 1 × *Shigella sonnei*,
UA		
6	47	13 × *Stenotrophomonas maltophilia*, 9 × *Shigella *species, 7 × γ proteobacterium, 2 × uncultured bacterium, 2 × uncultured *Veillonella *species, 1 × *Alcaligenes faecalis*, 1× *Agrobacterium *species, 1 × *Escherichia *species, 1 × *Streptococcus thermophilus*, 1 × *Flavobacterium mizutaii*, 1 × *Propionibacterium acnes*, 1 × uncultured β proteobacterium, 1 × uncultured bacterium, 1 × uncultured candidate division OP10 bacterium, 1 × *Pantoea ananatis*, 1 × *Shigella sonnei*, 1 × unidentiefied bacterium, 1 × uncultured *Flavobacterium *species, 1 × uncultured *Sphingobacterium *species.
7	30	8 × *Stenotrophomonas maltophilia*, 3 × *Shigella *species, 2 × *Comamonas testosteroni*, 2 × *Paenibacillus humicus*, 2 × *Ralstonia *species, 2 × uncultured bacterium, 2 × uncultured β proteobacterium, 2 × uncultured *Flavobacterium *species, 1 × *Burkholderia *species, 1 × *Bradyrhizobium elkanii*, 1 × Bacteroidetes bacterium, 1 × *Escherichia coli*, 1 × *Flavobacterium mizutaii*, 1 × γ proteobacterium, 1 × *Shigella sonnei*
8	43	*15 × Stenotrophomonas maltophilia*, 5 × *Shigella *species, 3 × *Escherichia *species, 3 × *Paenibacillus humicus*, 2 × uncultured bacterium, 2 × uncultured β proteobacterium, 1 × *Alcaligenes faecalis*, 1 × *Aranicola *species, 1 × *Bradyrhizobium elkanii*, 1 × *Flavobacterium mizutaii*, 1 × γ proteobacterium, 1 × *Ralstonia *species, 1 × *Staphylococcus pasteuri*, 1 × *Streptococcus mitis*, 1 × *Streptococcus pneumoniae*, 1 × *Streptococcus *species, 1 × uncultured candidate division OP10 bacterium, 1 × uncultured *Streptococcus *species, 1 × uncultured *Veillonella *species.
9	38	11 × *Aquabacterium commune*, 8 × *Shigella *species, 3 × *Aranicola *species, 3 × *Paenibacillus humicus*, 2 × *Alcaligenes faecalis*, 2 uncultured bacterium, 1 × β proteobacterium, 1 × *Comamonas testosteroni*, 1× *Escherichia *species, 1 × *Kocuria *species, 1 × *Pseudomonas *species, 1 × *Paracoccus *species, 1 × *Streptococcus thermophilus*, 1 × *Streptococcus *species, 1 × uncultured β proteobacterium
10	41	*13 × Stenotrophomonas maltophilia*, 4 × *Shigella *species, 3 × γ proteobacterium, 3 × *Shigella sonnei*, 1 × *Aranicola *species, 1 × *Alcaligenes faecalis*, 1× *Aeromonas *species, 1× *Burkholderia *species, 1 × *Bradyrhizobium elkanii*, 1 × *Comamonas testosteroni*, 1 × *Capnocytophaga sputigena *1 × *Enterococcus faecium*, 1× *Escherichia *species, 1 × *Enterobacter hormaechei*, 1 × *Pseudomonas poae*, 1 × *Pantoea ananatis*, 1 × *Paenibacillus humicus*, 1 × *Rhodococcus *species, 1 × *Ralstonia *species, 1 uncultured bacterium, 1 × uncultured γ proteobacterium, 1 × uncultured *Flavobacterium *species.
11	36	6 × *Stenotrophomonas maltophilia*, 5 × *Shigella *species, 4 × γ proteobacterium, 4 × *Ralstonia *species, 3 × *Escherichia coli*, 2 × *Bradyrhizobium elkanii*, 2 × *Comamonas testosteroni*, 2 × uncultured bacterium, 1 × *Achromobacter xylosoxidans*, 1 × *Alcaligenes faecalis*, 1 × Bacteroidetes bacterium, 1 × *Flavobacterium mizutaii*, 1 × *Rhizobium radiobacter*, 1 × *Rhodococcus *species, 1 × uncultured β proteobacterium, 1 × uncultured organism.
12	22	4 × *Shigella *species, 4 × *Stenotrophomonas maltophilia*, 3 × *Alcaligenes faecalis*, 2 × *Shigella sonnei*, 2 × uncultured β proteobacterium, 2 × uncultured bacterium, 1 × *Aminobacter aminovorans*, 1 × *Achromobacter xylosoxidans*, 1 × *Paenibacillus humicus*, 1 × *Ralstonia *species, 1 × uncultured organism.
13	37	12 × *Stenotrophomonas maltophilia*, 3 × *Shigella *species, 3 × uncultured bacterium, 2 × *Bacillus niacini*, 2 × *Ralstonia *species, 1 × β proteobacterium, 1 × *Bradyrhizobium japonicum*, 1 × *Burkholderia cepacia*, 1 × *Corynebacterium durum*, 1 × *Comamonas testosteroni*, 1 × *Flavobacterium mizutaii*, 1 × γ proteobacterium, 1 × uncultured β proteobacterium, 1 × uncultured *Sphingobacterium *species, 1 × *Mycobacterium aubagnense*, 1 × *Shigella sonnei*., 1 × *Sphingomonas *species, 1 × *Sphingomonas *species, 1 × *Pseudomonas poae*, 1 × *Pantoea ananatis*.
14	36	*10 × Stenotrophomonas maltophilia*, 7 × *Ralstonia *species, 3 × *Comamonas testosteroni*, 3 × uncultured bacterium, 2 × γ proteobacterium, 1 × *Escherichia *species, 1 × *Flavobacterium mizutaii*, 1 × *Methylomicrobium *species, 1 × *Paenibacillus humicus*, 1 × *Pantoea ananatis*, 1 × *Photorhabdus luminescens*, 1 × *Streptococcus pneumoniae*, 1 × uncultured *Sphingobacterium *species, 1 × uncultured *Flavobacterium *species, 1 × uncultured β proteobacterium, 1 × uncultured Firmicutes bacterium.
RA		
15	24	10 × *Stenotrophomonas maltophilia*, 3 × uncultured *Flavobacterium *species, 2 × *Comamonas testosteroni*, 2 × uncultured bacterium, 1 × *Aranicola *species, 1 × *Flavobacterium mizutaii*, 1 × *Paenibacillus humicus*, 1 × *Shigella *species, 1 × *Staphylococcus cohnii*, 1 × *Sphingobacterium thalpophilum*, 1 × uncultured *Sphingobacterium *species.
16	13	5 × uncultured β proteobacterium, 4 × uncultured *Flavobacterium *species, 2 × uncultured *Sphingobacterium *species, 1 × *Comamonas testosterone*, 1 × uncultured bacterium.
17	8	3 × *Escherichia *species, 2 × uncultured bacterium, 1 × *Alcaligenes faecalis*, 1 × *Comamonas testosteroni*, 1 × *Shigella *species.
OA		
18	18	4 × *Stenotrophomonas maltophilia*, 4 × *Shigella *species, 2 × *Flavobacterium mizutaii*, 2 × uncultured *Flavobacterium *species, 2 × uncultured *Sphingobacterium *species, 1 × *Acinetobacter junii*, 1 × *Escherichia coli O157*, 2 × uncultured bacterium.
19	16	5 × *Stenotrophomonas maltophilia*, 3 × *Shigella *species, 3 × uncultured bacterium, 1 × *Alcaligenes faecalis*, 1 × *Comamonas testosteroni*, 1 × *Flavobacterium mizutaii*, 1 × *Paenibacillus humicus*, 1 × *Ralstonia *species.
20	17	4 × *Stenotrophomonas maltophilia*, 3 × *Shigella *species, 3 × uncultured bacterium, 1 × *Achromobacter xylosoxidans*, 1 × *Alcaligenes faecalis*, 1 × *Bacillus cereus*, 1 × *Escherichia *species, 1 × *Rothia mucilaginosa*, 2 × uncultured β proteobacterium

OA = osteoarthritis; RA = rheumatoid arthritis; ReA = reactive arthritis; UA = undifferentiated arthritis.

**Table 3:** Bacterial species identified by sequencing of cloned 16S rDNA

Bacterium-derived DNA identified in SF samples	Number of patients in whom bacterial DNAs were detected	Accession number^a^	Length of the sequence^b^	% Similarity^c^
**Bacteria identified in ReA and UA patients (n = 69)**				
**Bacteria previously detected in arthritis**				
*Aeromonas *species	(1 UA)	[EMBL:AF099027]	1400	97.77
***Burkholderia ***species	(1 ReA + 1 UA)	[EMBL:AY769903]	1391	99.13
***Burkholderia ***species	(1 UA)	[EMBL:AY134849]	1388	99.71
***Burkholderia cepacia***	(1 UA)	[EMBL:AY946010]	1390	99.89
*Escherichia coli*	(1 ReA)	[EMBL:CP000243]	1400	99.14
*Escherichia coli*	(2 ReA)	[EMBL:U00096]	1400	100.00
*Escherichia coli*	(1 ReA + 2 UA)	[EMBL:V00348]	1400	99.50
***Methylomicrobium ***species	(1 UA)	[EMBL:AF194538]	1380	98.83
*Propionibacterium acnes*	(5 ReA + 1 UA)	[EMBL:AB108477]	1386	99.05
*Pseudomonas *species	(1 ReA)	[EMBL:DQ213044]	1386	99.06
*Pseudomonas *species	(1 ReA)	[EMBL:AY014811]	1395	99.06
*Pseudomonas *species	(1 UA)	[EMBL:AM409368]	1396	99.21
*Paracoccus *species	(1 UA)	[EMBL:AY745834]	1320	98.92
*Rhodococcus *species	(2 UA)	[EMBL:AF420412]	1374	97.29
*Shigella sonnei*	(5 ReA + 5 UA)	[EMBL:CP000038]	1400	99.71
*Shigella sonnei*	(1 UA)	[EMBL:X96964]	1389	99.78
*Sphingomonas *species	(1 UA)	[EMBL:AB110635]	1341	99.85
*Sphingomonas *species	(1 UA)	[EMBL:AF385529]	1341	100.00
*Streptococcus *species	(1 UA)	[EMBL:AF316593]	1400	98.55
*Streptococcus *species	(1 UA)	[EMBL:AF385523]	1400	99.28
*Streptococcus mitis*	(1 ReA)	[EMBL:AJ295848]	1400	97.92
*Streptococcus mitis*	(1 ReA)	[EMBL:AJ617805]	1322	98.71
*Streptococcus mitis*	(1 ReA)	[EMBL:AF003929]	1396	99.36
*Streptococcus mitis*	(1 ReA + 1 UA)	[EMBL:AY518677]	1404	99.86
*Streptococcus pneumoniae*	(2 UA)	[EMBL:AM157442]	1400	98.64
*Streptococcus thermophilus*	(2 UA)	[EMBL:AY188354]	1397	99.57
**Bacteria not previously detected in arthritis but detected in human infection**				
***Agrobacterium ***species	(2 ReA + 1 UA)	[EMBL:AY775177]	1344	99.40
*Bradyrhizobium elkanii*	(2 ReA + 4 UA)	[EMBL:AY904749]	1347	99.40
***Capnocytophaga sputigena***	(1 UA)	[EMBL:AF133536]	1378	98.96
*Corynebacterium durum*	(1 UA)	[EMBL:AF537593]	1391	99.58
***Delftia acidovorans***	(1 ReA)	[EMBL:AB020186]	1309	97.63
*Enterococcus faecium*	(1 UA)	[EMBL:EF533988]	1396	99.42
*Enterobacter hormaechei*	(1 UA)	[EMBL:AY995561]	1400	99.70
***Kocuria ***species	(1 UA)	[EMBL:AY864652]	1387	99.85
***Mycobacterium aubagnense***	(1 UA)	[EMBL:AY859683]	1372	99.27
***Pantoea ananatis***	(3 ReA + 4 UA)	[EMBL:DQ133546]	1400	98.80
***Photorhabdus luminescens***	(1 UA)	[EMBL:AY444555]	1333	99.85
*Pseudomonas poae*	(2 UA)	[EMBL:AJ492829]	1386	98.99
***Rhizobium radiobacter***	(1 UA)	[EMBL:AJ389902]	1300	99.67
***Staphylococcus pasteuri***	(1 UA)	[EMBL:AJ717376]	1411	99.86
**Bacteria not previously detected in humans**				
*Aquabacterium commune*	(1 UA)	[EMBL:AF035054]	1416	99.70
***Aminobacter aminovorans***	(1 UA)	[EMBL:AJ011759]	1344	98.72
***Bacillus niacini***	(1 UA)	[EMBL:AB021194]	1400	99.14
*Bradyrhizobium japonicum*	(1 UA)	[EMBL:AB072418]	1200	97.81
***Diaphorobacter ***species	(1 ReA)	[EMBL:DQ294626]	1387	99.78
***Pelomonas saccharophila***	(1 ReA)	[EMBL:AB021407]	1381	99.42
***Paenibacillus ***species	(1 ReA)	[EMBL:AM162345]	1385	99.35
***Sphingomonas faeni***	(1 ReA)	[EMBL:AJ429239]	1340	97.82
**Uncultured bacteria**				
Bacteroidetes bacterium	(2 UA + 1 ReA)	[EMBL:AY395022]	1394	96.11*
Bacteroidetes bacterium	(1 ReA)	[EMBL:DQ195837]	1300	98.39
β Proteobacterium	(2 ReA + 2 UA)	[EMBL:AY162033]	1396	99.57
γ Proteobacterium	(4 ReA + 6 UA)	[EMBL:AY162042]	1399	99.00
γ Proteobacterium	(1 ReA + 4 UA)	[EMBL:AY162068]	1400	99.50
swine manure bacterium	(1 ReA)	[EMBL:AY167969 ]	1395	99.21
Uncultured α proteobacterium	(1 ReA)	[EMBL:AF445680]	1342	97.00
Uncultured Bacteroidetes bacterium	(2 ReA)	[EMBL:AY921801]	1384	97.04
Uncultured bacterium	(2 UA)	[EMBL:AY958813]	1388	99.88
Uncultured β proteobacterium	(1 ReA + 1 UA)	[EMBL:AF445700]	1372	99.78
Uncultured β proteobacterium	(1 UA)	[EMBL:DQ316806]	1391	97.46
Uncultured candidate division OP10 bacterium	(2 UA)	[EMBL:AF418946]	1362	90.29*
Uncultured Firmicutes bacterium	(1 UA)	[EMBL:EF071401]	1400	99.57
Uncultured γ Proteobacterium	(1 UA)	[EMBL:AF324537]	1396	99.96
Uncultured γ Proteobacterium	(1 ReA)	[EMBL:AJ318146]	1400	97.63
Uncultured γ Proteobacterium	(1 ReA)	[EMBL:AY770720]	1393	96.91*
Uncultured Methylococcaceae bacterium	(1 ReA)	[EMBL:EF019533]	1398	97.55
Uncultured organism	(2 UA)	[EMBL:DQ395839]	1392	99.85
Uncultured soil bacterium	(1 ReA)	[EMBL:DQ297948]	1340	99.85
Uncultured *Streptococcus *species	(1 UA)	[EMBL:AY256519]	1400	99.21
Uncultured *Veillonella *species	(2 UA)	[EMBL:AM157449]	1400	99.37
**Bacteria identified in control group (RA and OA patients; n = 10)**				
**Bacteria previously detected in arthritis (in joint)**				
***Acinetobacter junii***	(1 OA)	[EMBL:AB101444]	1387	99.28
***Bacillus cereus***	(1 OA)	[EMBL:AB247137]	1399	98.36
***Rothia mucilaginosa***	(1 OA)	[EMBL:DQ409140]	1375	98.69
***Staphylococcus cohnii***	(1 RA)	[EMBL:AJ717378]	1399	99.57
**Bacteria not previously detected in arthritis but detected in human infection**				
***Escherichia coli O157***	(1 OA)	[EMBL:AE005174]	1394	97.56
**Bacteria not previously detected in humans**				
***Sphingobacterium thalpophilum***	(1 RA)	[EMBL:AJ43817]	1388	94.02*
**Uncultured bacteria**				
Uncultured bacterium	(1 OA)	[EMBL:DQ800655]	1394	97.99
Uncultured bacterium	(1 RA)	[EMBL:AY958855]	1388	99.78
Uncultured bacterium	(1 RA)	[EMBL:AY958896]	1380	99.57
Uncultured bacterium	(1 RA + 1 OA)	[EMBL:DQ818800]	1398	99.57
**Common bacteria^d ^(n = 20)**				
**Bacteria previously detected in arthritis**				
*Achromobacter xylosoxidans*	(1 ReA + 2 UA+ 1 OA)	[EMBL:AF439314]	1389	99.50
*Alcaligenes faecalis*	(2 ReA + 6 UA + 2 OA + 1 RA)	[EMBL:AY548384]	1395	99.70
*Comamonas testosterone*	(1 ReA + 4 UA +1 OA + 3 RA)	[EMBL:AB007996]	1390	99.35
*Comamonas testosterone*	(2 ReA + 3 UA + 1 OA)	[EMBL:M11224]	1390	98.05
*Escherichia *species	(3 ReA + 5 UA + 1 OA + 1 RA)	[EMBL:DQ337503]	1400	99.86
*Flavobacterium mizutaii*	(4 ReA + 6 UA + 2 OA + 1 RA)	[EMBL:AJ438175]	1385	94.00*
*Shigella *species	(5 ReA + 8 UA + 3 OA + 2 RA)	[EMBL:DQ337523]	1399	99.70
*Stenotrophomonas maltophilia*	(5 ReA + 8 UA + 3 OA + 1 RA)	[EMBL:AJ293470]	1396	99.75
*Stenotrophomonas maltophilia*	(5 ReA + 8 UA + 3 OA + 1 RA)	[EMBL:AB294557]	1396	99.86
**Bacteria not previously detected in arthritis but detected in human infection**				
***Aranicola ***species	(4 ReA + 3 UA + 1 RA)	[EMBL:AM398227]	1400	99.64
*Ralstonia *species	(3 ReA + 7 UA + 1 OA)	[EMBL:AB045276]	1387	99.86
**Bacteria not previously detected in humans**				
***Paenibacillus humicus***	(4 ReA + 6 UA +1 OA + 1 RA)	[EMBL:AM411528]	1399	98.60
**Uncultured bacteria**				
Uncultured bacterium	(4 ReA + 4 UA + 1 OA)	[EMBL:DQ818781]	1394	98.90
Uncultured bacterium	(5 ReA + 4 UA + 2 OA + 2 RA)	[EMBL:AY838480]	1389	94.00*
Uncultured bacterium	(2 ReA + 3 UA + 1 OA + 2 RA)	[EMBL:AB076874]	1389	94.00*
Uncultured bacterium	(1 ReA +1 OA)	[EMBL:AY838458]	1382	99.64
Uncultured bacterium	(1 UA + 1 OA)	[EMBL:DQ824599]	1398	99.57
Uncultured β proteobacterium	(4 ReA + 6 UA + 1 OA + 1 RA)	[EMBL:DQ366010]	1384	99.71
Uncultured *Flavobacterium *species	(3 ReA + 5 UA + 1 OA + 2 RA)	[EMBL:DQ366085]	1300	97.20
Uncultured *Sphingobacterium *species	(1 ReA + 3 UA + 1 OA + 2 RA)	[EMBL:AB076874]	1389	94.16*

Number in brackets after species names indicate the number of patient set from whom bacteria were detected. ^a^Accession number of the bacterial species in the EMBL database. ^b^Length of alignment on which the 16S rDNA inserted sequence and the corresponding sequence in the database are similar. ^c^In the '% similarity' column, asterisks indicate highlight instances where the % similarity is below 97%. ^d^The 'Common bacteria' row shows the bacteria identified in ReA, UA, RA, and OA patients.

Bacterial species detected only in SF samples and not in ST samples from our previous study [15], are indicated in bold.

OA = osteoarthritis; RA = rheumatoid arthritis; ReA = reactive arthritis; SF = synovial fluid; UA = undifferentiated arthritis.

### Bacterial 16S rDNA sequences identified in synovial fluid samples

Each patient's SF samples contained a diverse range of bacterial DNA-related species (Table 2). The majority of pathogens across all groups were identified as *Shigella *species and *Stenotrophomonas maltophilia*. DNA from a total of 69 various individual bacterial species were detected in SF samples from ReA and UA patients, whereas only 10 different bacterial DNA were found in SF samples from RA and OA patients. In addition, DNA from 20 bacterial species was detected in both the study and the control groups.

In ReA and UA patients, apart from *Shigella sonnei *DNA, there was no other DNA-derived bacteria so far described to trigger ReA. There were sequences from commensal bacteria, in particular those from the skin or the intestinal tract (*Propionibacterium acnes*, *Escherichia coli*, and other coliform bacteria). We also detected additional species previously assigned to the *Pseudomonas *genus such as DNA of *Pseudomonas poae*, *Delftia acidovorans*, and *Burkholderia cepacia*. A detailed sequence analysis of the PCR-positive samples of ReA and UA patients revealed a number of DNA of bacteria that have previously been described in human infections but not in arthritis, including *Rhizobium radiobacter*, *Pantoea ananatis*, and *Capnocytophaga sputigena*. Except for *P. ananatis*, these DNA sequences were observed in only one individual (Table 3)

DNA products from environmental bacteria previously detected in arthritis, such as *Achromobacter xylosoxidans*, *Alcaligenes faecalis*, and *Flavobacterium mizutaii *were commonly found in both the study and control groups. DNA of *Aranicola *species bacterium rarely described in humans but not associated with arthritis was common in patients with ReA, UA, and RA and was detected in more than one case (Table 3).

Some clones fell into environmental species not previously reported in human infections including DNA of *Paenibacillus humicus*, *Bacillus niacini*, and *Diaphorobacter *species, and other bacteria, which have not yet been cultured (Table 3).

DNA from the candidate division OP10 bacterium was detected in SF samples of only two patients with UA. These bacterial DNA sequences have not been previously characterized by rDNA sequencing because they exhibit less than 97% similarity to known database sequences. We could find no clear association between the presence of a particular bacterial DNA and clinical symptoms.

## Discussion

In the present study we used broad-range PCR amplification, cloning, and sequencing of the full-length 16S rDNA from a wide variety of bacterial species in the SF samples of all patients studied. Only a few studies have been conducted to detect and identify the bacterial DNA communities in SF samples of patients with ReA, UA, and others arthropathies. In these studies, only short fragments of DNA were amplified allowing solely the determination of the bacterial genus but not the identification of the species level. This is, to our knowledge, the first study using the full-length 16S rRNA gene as a target for broad-spectrum PCR to detect bacterial DNA in SF samples allowing the identification of the species level.

Sequence analysis of the PCR-positive samples revealed the presence of a wide spectrum of bacterial DNA in SF samples of all studied patients. False positivity due to contamination poses a problem when broad-range PCR targeting the 16S rRNA is used [20-22]. However, our recently published study outlines extensive measures, which were also taken in the present study in order to avoid any contamination [15]. From a practical point of view, it is important to notice that we did not surgically incise the skin at the site of the aspiration thus avoiding contamination by the skin flora but our results are rather similar to those obtained by others who took this precaution [14]. PCR and extraction controls consistently yielded negative results indicating that the PCR products detected in positive samples are most likely derived from the bacterial rRNA genes actually present in SF cells. In addition, the sequences obtained varied between patients and DNA from several organisms has also been identified in arthritic human joints in independent laboratories [12-14,17,18], suggesting that their presence is unlikely to be a consequence of contamination.

The most common sequences of species found in SF samples of all patients (e.g. *A. xylosoxidans*, *A. faecalis, F. mizutaii*, and *S. maltophilia*) were also seen in the previous study [15], except the DNA from *Aranicola *species implying that they might be opportunistic colonizers of inflamed joints. *S. maltophilia *DNA was identified most frequently. This organism is an opportunistic pathogen and has previously been detected in arthritic knee joints [12,15,17]. Of note, other bacterial DNA have been described in human infections but not so far in arthritis were identified in the SF samples of our arthritic patients. Some of them are detected only in SF samples but not in their matched ST samples, such as *R. radiobacter *and *Pantoae ananatis*. Recently, we reported that DNA derived from uncultured bacteria and from environmental organisms that have not been previously detected in human samples could also be demonstrated in ST samples [15]. Similarly, in SF samples such bacterial DNA was detected including *Aminobacter aminovorans, B. niacini, Diaphorobacter *species, and uncultured candidate division OP10 bacterium. Several studies have also detected unsuspected uncultured and/or cultured bacteria not considered as human pathogens in arthritic joints but they were found by sequencing short DNA fragments [12-14,16]. Thus, the identification of such bacterial species after cloning and near-full length 16S DNA sequencing might be of interest and should be pursued. However, their presence in the joint can not provide definite evidence of their replication or a functional role in arthritis.

Our results also confirmed the presence of *E. coli *sequences in SF samples as previously found in ST samples of arthritis patients [15]. This could indicate the ability of *E. coli *DNA to colonize inflamed joints; the gut in different patients would be expected to contain a material derived from a range of *E. coli *'subspecies' [23-25].

Our analysis of SF samples and their matched ST samples confirmed a wide spectrum of bacterial DNA-related species detected in each individual patient. Accordingly, a significant correlation was found between the diversity of bacterial species detected in SF and matched ST at the patient level (r = 0.522, *P *= 0.018). However, they revealed a different profile in regard to their known potential of triggering ReA in either SF or ST samples. Thus, the *Shigella flexneri *sequences were not detected in any of the SF samples whereas *S. sonnei *16 rDNA sequences were detected more frequently in SF samples of five ReA and five UA patients as compared with the ST samples of one ReA and one UA patient [15]. DNA of *Shigella *species was also prevalent in SF samples as demonstrated previously in ST samples [15], but it was more frequently detected in SF samples (five ReA, eight UA, two RA, and three OA in SF vs two ReA, four UA, two RA, and one OA in ST samples). Although *S. sonnei *and *Shigella *species were detected more frequently in SF than in ST samples, the difference was statistically not significant.

We detected DNA from *Shigella *species in our cohort of patients with various forms of arthritis and *S. sonnei*, known to trigger ReA, only in ReA and UA samples. *Shigella *DNA positive patients had no clinical signs of previous intestinal infection with an enteric organism. These patients may have been asymptomatic, or the preceding gastrointestinal symptoms may have been mild and overlooked by the patients [3]. It is possible that enteric organisms may migrate from asymptomatic primary sites of the infection to the synovial compartment [3]. Most *Shigella *ReA caused by *S. sonnei *are sporadic cases [26]. The most recent published case of *S. sonnei *related ReA was attributed to sexual transmission of the pathogen [27]. In our study, we detected *S. sonnei *DNA in five ReA patients presenting with an urogenital infection, which is consistent with the possibility that this species could be related to sexual transmission.

A composition of a mixture of bacterial nucleic acids was common in our cohort of patients with various forms of arthritis, as has been described in previous studies [13-15,17,18]. The bacterial DNA might be incorporated by macrophages, which are disseminated by the circulation and reach the joint due to an increased cellular recruitment. As opposed to a single organism, such mixtures may increase the risk of triggering an immune response finally culminating in synovitis. However, genetic susceptibility factors of the host are also playing an important role particularly in persistent infections [10,28]. As mixtures of bacterial nucleic acids are also detectable in the SF samples from patients with RA and OA, we cannot exclude that this may represent a normal 'background' phenomenon not necessarily causing synovitis [10,17]. Another limitation of our study lies in the fact that we could not detect *Chlamydia trachomatis *DNA or other common bacteria known to trigger posturethritic arthritis.

## Conclusions

Our study provides a valuable overall picture of the bacterial DNA environment present in the SF of the actively inflamed joints of arthritis patients. Characterization of the DNA reveals a wide spectrum of organisms so far not known to be present in human infections, not known to be present in inflamed joints of arthritis patients, and not known to trigger ReA. There is also a differential bacterial colonisation and/or infection of SF and ST samples because the analysis of SF can identify a number of bacterial DNA-related species, which have not been detected in ST samples as studied earlier [15] and has helped to confirm that the composition of bacterial DNA may change over time in joint cavity.

Accordingly, the analysis of SF or ST samples from different arthropathies patients by broad-range PCR is essentially capable of characterising the bacterial DNA environment present in joint cavity. As synovial biopsy is a difficult act, SF is well practical for such purpose.

## Abbreviations

OA: osteoarthritis; PCR: polymerase chain reaction; RA: rheumatoid arthritis; ReA: reactive arthritis; SF: synovial fluid; ST: synovial tissue; UA: undifferentiated arthritis.

## Competing interests

The authors declare that they have no competing interests.

## Authors' contributions

MS performed the experimental work, analyzed the data, and wrote the manuscript. RG conceived of the study, performed the design and coordination of the study, analyzed the data, and revised the manuscript. HF, MY, SB, NB, and SS made pathological diagnosis, conducted sampling procedures, and performed clinical and rheumatological data analyses. BJ and JS participated in the design and coordination of the study, and drafted the manuscript. MR has assisted in writing the manuscript. AH and AS analyzed microbiological and sequencing data, and revised the manuscript. All authors read and approved the final manuscript.
